# The proline–arginine repeat protein linked to C9-ALS/FTD causes neuronal toxicity by inhibiting the DEAD-box RNA helicase-mediated ribosome biogenesis

**DOI:** 10.1038/s41419-018-1028-5

**Published:** 2018-09-24

**Authors:** Hiroaki Suzuki, Yoshio Shibagaki, Seisuke Hattori, Masaaki Matsuoka

**Affiliations:** 1 Department of Pharmacology, Tokyo Medical University, 6-1-1 Shinjuku, Shinjuku-ku, Tokyo 160-8402 Japan; 2 Division of Biochemistry, School of Pharmaceutical Sciences, Kitasato University, 5-9-1 Shirokane, Minato-ku, Tokyo 108-8641 Japan; 3 Department of Dermatological Neuroscience, Tokyo Medical University, 6-1-1 Shinjuku, Shinjuku-ku, Tokyo 160-8402 Japan

## Abstract

A GGGGCC repeat expansion in the *C9ORF72* gene has been identified as the most common genetic cause of amyotrophic lateral sclerosis and frontotemporal dementia. The repeat expansion undergoes unconventional translation to produce dipeptide repeat (DPR) proteins. Although it has been reported that DPR proteins cause neurotoxicity, the underlying mechanism has not been fully elucidated. In this study, we have first confirmed that proline–arginine repeat protein (poly-PR) reduces levels of ribosomal RNA and causes neurotoxicity and found that the poly-PR-induced neurotoxicity is repressed by the acceleration of ribosomal RNA synthesis. These results suggest that the poly-PR-induced inhibition of ribosome biogenesis contributes to the poly-PR-induced neurotoxicity. We have further identified DEAD-box RNA helicases as poly-PR-binding proteins, the functions of which are inhibited by poly-PR. The enforced reduction in the expression of DEAD-box RNA helicases causes impairment of ribosome biogenesis and neuronal cell death. These results together suggest that poly-PR causes neurotoxicity by inhibiting the DEAD-box RNA helicase-mediated ribosome biogenesis.

## Introduction

Amyotrophic lateral sclerosis (ALS) is an incurable motor neuron disease, characterized by a selective loss of both upper and lower motor neurons^1,2^. Frontotemporal dementia (FTD) is a type of dementia, characterized by the degeneration of the frontal and temporal lobes^3,4^. Although it has been recognized that they have common clinical and pathological features^1,5^, the underlying pathogenic mechanism remains unclear.

A GGGGCC (G4C2) hexanucleotide repeat expansion within the first intron and the promoter of the chromosome 9 open reading frame 72 (*C9ORF72*) gene has been identified as the most frequent genetic cause of ALS and FTD (C9-ALS/FTD)^6,7^. Three hypotheses have been proposed for the pathogenesis underlying C9-ALS/FTD: (1) the loss-of-function of C9ORF72 that is caused by the G4C2 repeat expansion-mediated reduction in the expression of C9ORF72 protein, (2) the gain-of-toxic-function of RNA foci that is caused by the load of expanded G4C2 transcripts, and (3) the accumulation of dipeptide repeat (DPR) proteins, which are produced by the G4C2 repeat-associated non-ATG (RAN) translation and toxic to neurons^8^. The RAN translation generates five distinct DPR proteins from six bidirectional transcribed open reading frames of the repetitive sequence: poly-glycine–alanine (GA), poly-glycine–arginine (GR), poly-proline–arginine (PR), poly-proline–alanine (PA), and poly-glycine–proline (GP)^9–12^. It has been shown that all these DPR proteins are really expressed in the central nervous systems of patients with C9-ALS/FTD and the enforced expression of any of DPR proteins likely causes neuronal toxicity in vitro and in vivo^13^. Currently, however, the relative contribution of each DPR protein to the C9-ALS/FTD pathogenesis and the mechanism underlying the DPR protein-induced neuronal toxicity have not been fully elucidated.

RNA helicases are highly conserved enzymes that play essential roles in most aspects of mRNA metabolism and ribosome biogenesis^14–16^. They remodel RNA and ribonucleoprotein complexes mainly by unwinding RNA duplexes in an ATP-dependent fashion. Besides unwinding activity, several RNA helicases have wide-range activities related to the RNA metabolism^14^. DEAD-box RNA helicases 5 (DDX5) acts as a potent transcriptional co-activator of the tumor suppressor p53, in which the helicase activity is not required^17^. DDX21 shows ATP-independent strand-annealing activity during RNA folding^18^. Thus, as their activities are essential in various cellular processes, the malfunction of RNA helicases is likely linked to human diseases such as cancer and neurological disorders^19^. For example, it has been reported that several mutations in *SETX*, a gene encoding Senataxin, which is structurally classified as a DNA/RNA helicase, cause autosomal dominant juvenile ALS (ALS4)^20^. DEAD-box RNA helicases constitute the largest family of RNA helicases^15^. They contain an Asp–Glu–Ala–Asp (DEAD) motif in the middle region. Forty DEAD-box RNA helicases have now been identified in humans^14^. Generally, the functions of most RNA helicases have been insufficiently characterized.

In this study, using an in vitro cell-based assay, we first reproduced that the enforced expression of poly-GA, poly-GR, or poly-PR caused neuronal cell death. Considering that poly-PR caused the most prominent neuronal toxicity in our assay, we next focused on the analysis of the poly-PR-mediated neuronal cell death. We first confirmed that the overexpression of poly-PR caused neurotoxicity and reduced levels of ribosomal RNA (rRNA)^21,22^ and then found that the poly-PR-induced neuronal cell death is restored by the acceleration of ribosome biogenesis. We further found that poly-PR interacted with multiple DEAD-box RNA helicases and inhibited the function of at least one of them, and that the reduction in the levels of some RNA helicases resulted in both the decrease in rRNA levels and the increase in neuronal cell death. Altogether, these results suggest that poly-PR causes neuronal toxicity by inhibiting the DEAD-box RNA helicase-mediated ribosome biogenesis.

## Results

### Characterization of intracellular DPR proteins

**Fig. 1:**
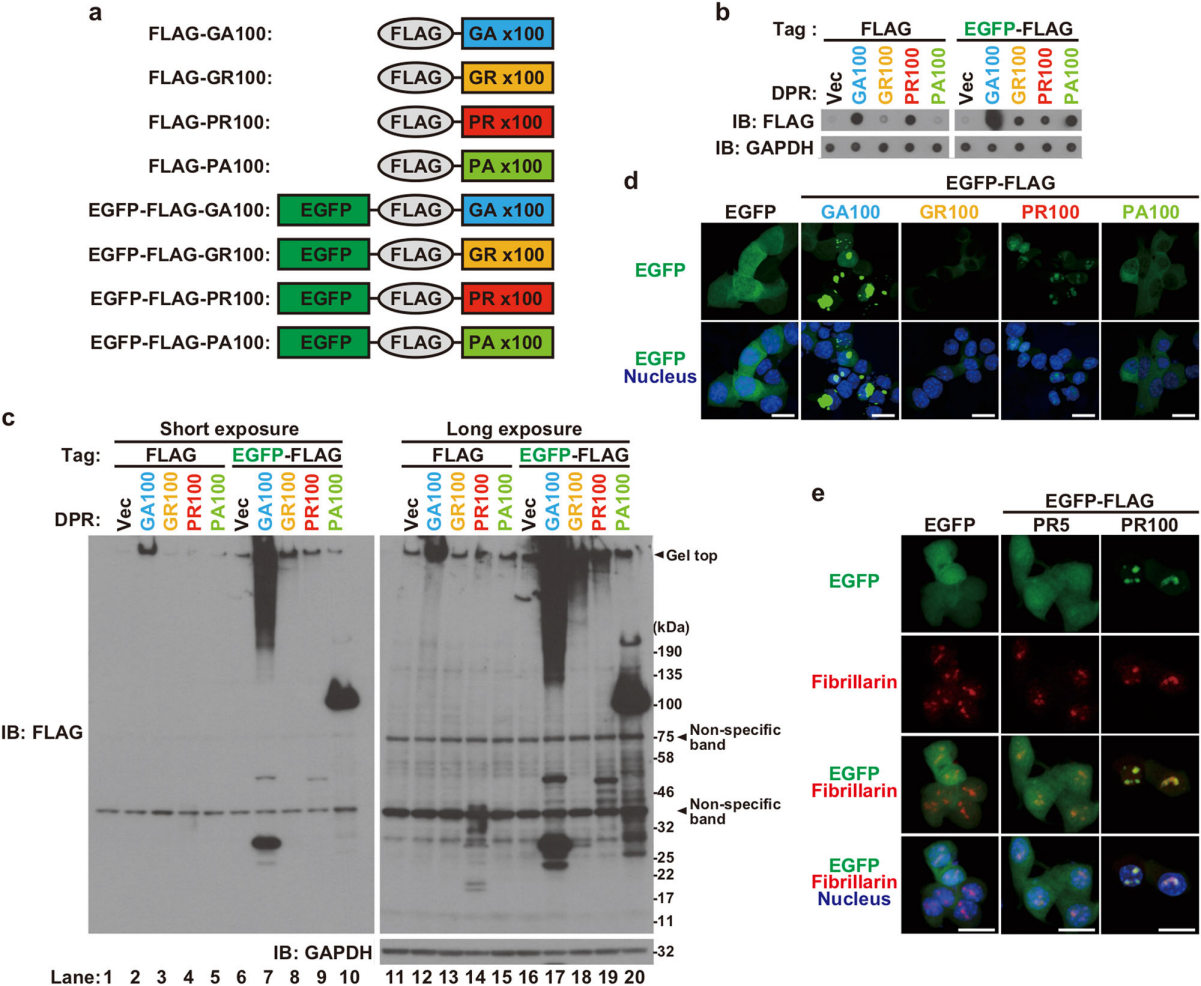
Characterization of dipeptide repeat (DPR) proteins. **a** Schematic illustrations of N-terminal FLAG- or EGFP-FLAG-tagged DPR proteins. All constructs contain 100 repeats of dipeptide. **b**, **c** NSC-34 cell lysates overexpressing FLAG- or EGFP-FLAG-DPR were subjected to dot blotting analysis (**b**) and immunoblotting (IB) (**c**) using FLAG antibody. **d** NSC-34 cells overexpressing EGFP-FLAG-DPR (green) were fixed and stained with DAPI (blue). Scale bar: 20 μm. **e** NSC-34 cells overexpressing EGFP, EGFP-FLAG-PR5, or EGFP-FLAG-PR100 (green) were fixed and immunostained with fibrillarin (red). Nuclei were stained with Hoechst33258 (blue). Scale bar: 20 μm

We chemically synthesized four cDNAs encoding a 100-repeat of each DPR protein (DPR100) with an N-terminal FLAG tag, named FLAG-GA100, FLAG-GR100, FLAG-PR100, and FLAG-PA100 (Fig. 1a). The cDNAs contain a single open reading frame composed of a non-G4C2 codon of each DPR protein. It has been reported that the 100-repeat length of dipeptide is sufficient to cause neuronal toxicity and pathological features observed in C9-ALS/FTD in vitro and in vivo^23^. Unfortunately, a cDNA encoding FLAG-GP100 was incapable of being synthesized, due to an undetermined reason. We then transfected a mammalian expression vector that encodes a FLAG-DPR100 into NSC-34 motor neuron cells and analyzed their expression using both dot and western blotting analysis. Immunostaining with a FLAG antibody showed strong expression of FLAG-GA100 in both dot (Fig. 1b) and western blotting analysis in which a clear band was observed at the gel top and a faint smear was recognized in the lane under the gel top (Fig. 1c, lane 2 by short exposure and lane 12 by long exposure). Very weak expression of FLAG-GR100 was observed by dot blotting analysis (Fig. 1b), whereas no apparent expression of FLAG-GR100 was recognized by western blotting analysis (Fig. 1c, lane 13 by long exposure). The strong expression of FLAG-PR100 was observed by dot blotting analysis (Fig. 1b) and several faint bands, possibly derived from FLAG-PR100, were recognized within the molecular-weight-marker range of 17–46 kDa by the western blotting analysis (Fig. 1c, lane 14 by long exposure). No apparent expression of FLAG-PA100 was observed either by dot or western blotting analysis (Fig. 1b, c, lane 15 by long exposure). Discrepancy in the levels of expression, estimated by dot and western blotting analysis, suggested that the estimation of the total levels of DPR proteins by the dot blotting analysis is more correct than that by the western blotting analysis and that the chemical characteristics of a DPR protein lowers transfer efficiency to the polyvinylidene fluoride membrane during the western blotting analysis.

We next constructed expression vectors encoding EGFP-FLAG-tagged DPR100, in which cDNAs encoding FLAG-tagged DPR proteins were inserted into an N-terminally EGFP-tagged mammalian expression vector (Fig. 1a). The dot blotting analysis showed that all these proteins were expressed (Fig. 1b). By the western blotting analysis, we further found that the expression of EGFP-FLAG-GA100 resulted in several bands with apparent molecular weights of 25–50 kDa in addition to a high-molecular-weight smear (Fig. 1c, lane 7 by short exposure and lane 17 by long exposure). EGFP-FLAG-GR100 showed a high-molecular-weight smear (Fig. 1c, lane 8 by short exposure and lane 18 by long exposure). Expression of EGFP-FLAG-PR100 generated several bands with apparent molecular weights of 40–50 kDa and a high-molecular-weight smear (Fig. 1c, lane 19 by long exposure). Expression of EGFP-FLAG-PA100 showed a prominent band with a molecular weight of 100 kDa in addition to multiple bands with apparent molecular weights of 25–200 kDa (Fig. 1c, lane 10 by short exposure and lane 20 by long exposure). Unusual chemical characteristics of each DPR100 as well as its post-translational modification and its property to make a complex with other proteins may affect its electrophoretic mobility on SDS-PAGE and cause inefficient transfer to the membrane during western blotting analysis.

Using immunocytochemical analysis, we also found that the majority of EGFP-FLAG-GA100 looked like large aggregates in the nucleus and cytoplasm. EGFP-FLAG-GR100 and EGFP-FLAG-PA100 were diffusely localized in the cytoplasm. The majority of EGFP-FLAG-PR100 showed large dot-like localization in the nucleus. EGFP-FLAG-PR100 also showed a diffused nuclear and cytoplasmic localization (Fig. 1d). The large dot-like nuclear localization of EGFP-FLAG-PR100 (Fig. 1d) suggests that EGFP-FLAG-PR100 localizes in some intranuclear organelle or macromolecular complexes. As expected, immunocytochemical analysis showed that EGFP-FLAG-PR100, but not EGFP-FLAG-PR5, which consists of a 5-repeat PR, co-localized with fibrillarin, a nucleolus marker (Fig. 1e). This finding has already been reported by independent groups using cDNAs encoding different-length poly-PR^13,24^.

### The expression of DPR proteins causes neuronal toxicity

**Fig. 2:**
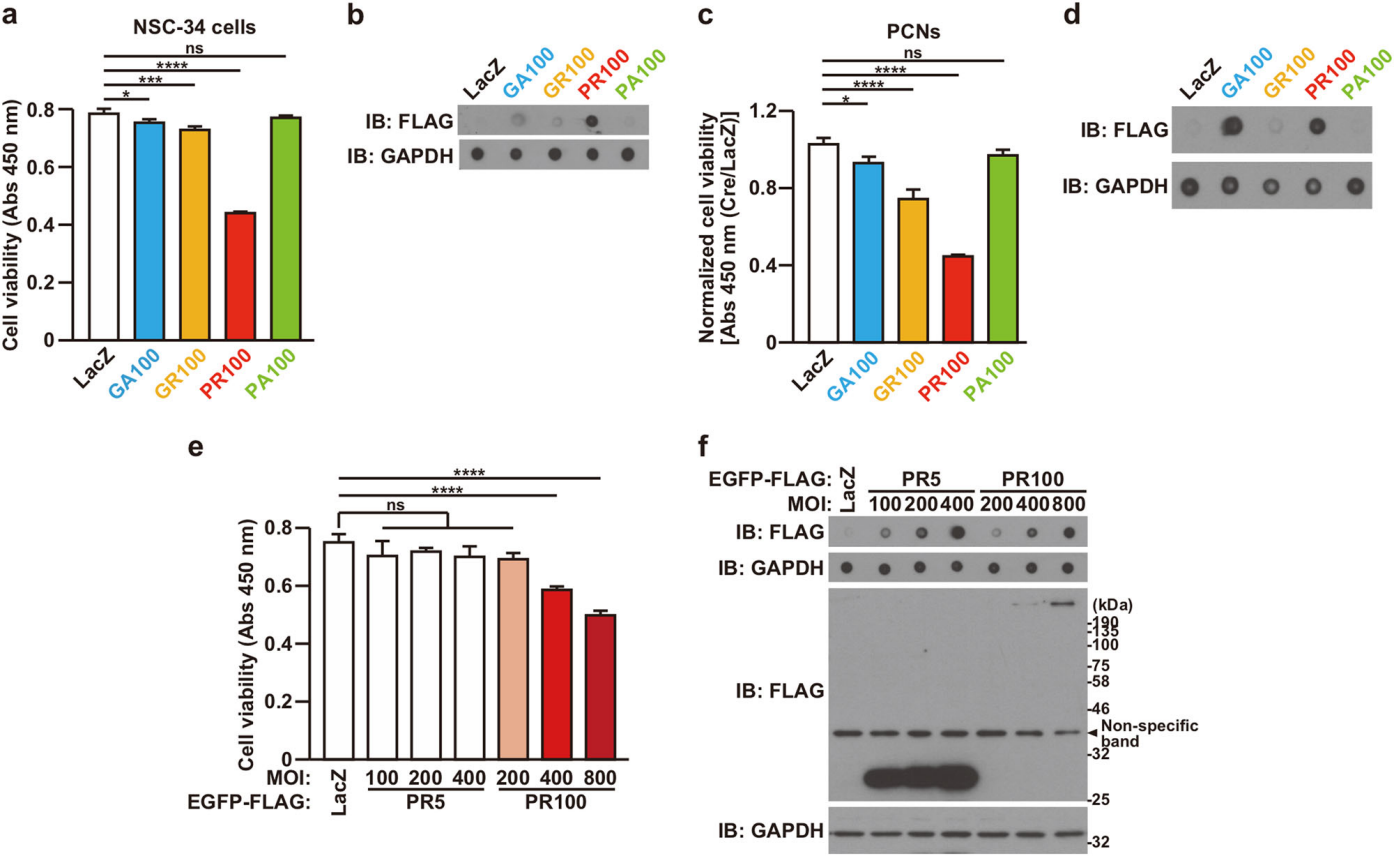
GA100, GR100, and PR100 cause neuronal toxicity. **a**, **b** NSC-34 cells were infected with adenovirus encoding LacZ or FLAG-DPR at an MOI of 800 together with adenovirus encoding Cre-recombinase at an MOI of 40. At 48 h after the infection, the cell viability was detected by WST-8 assay (**a**) and the cell lysates were subjected to dot blotting analysis using FLAG antibody (**b**). Means ± SD, *N* = 3 biological replicates. Statistical analysis was determined by one-way ANOVA followed by Dunnett’s multi comparisons test. **c**, **d** PCNs were infected with adenovirus encoding LacZ or FLAG-DPR at an MOI of 200 together with adenovirus encoding LacZ or Cre-recombinase at an MOI of 40. At 120 h after the infection, the cell viability was detected by WST-8 assay (**c**) and the cell lysates were subjected to dot blotting analysis using FLAG antibody (**d**). For normalization, the value of absorbance obtained from Cre-recombinase-infected cells was divided by the average of absorbance obtained from LacZ-infected cells. Means ± SD, *N* = 3 biological replicates. Statistical analysis was determined by one-way ANOVA followed by Dunnett’s multi comparisons test. **e**, **f** NSC-34 cells were infected with adenovirus encoding LacZ, EGFP-FLAG-PR5, or EGFP-FLAG-PR100 at MOIs of 100-800 together with adenovirus encoding Cre-recombinase at an MOI of 40. To keep the total MOIs of adenoviruses constant, appropriate MOIs of LacZ-encoding adenovirus were added for each infection. At 48 h after the infection, the cell viability was detected by WST-8 assay (**e**) and the cell lysates were subjected to immunoblotting (IB) and dot blotting analysis using FLAG antibody (**f**). Means ± SD, *N* = 3 biological replicates. Statistical analysis was determined by one-way ANOVA followed by Dunnett’s multi comparisons test

To express DPR100 in cells with a high efficiency, we next constructed DPR protein-encoding adenoviruses and examined whether a DPR100 causes neuronal toxicity to NSC-34 cells and mouse primary cortical neurons (PCNs). LacZ-encoding adenovirus was used as negative control, which does not show any cytotoxicity^25^. The expression of all FLAG-DPR100 except FLAG-PA100 reduced cell viability in both NSC-34 cells (Fig. 2a) and PCNs (Fig. 2c). Due to insufficient levels of overexpression (Fig. 2b, d), it could not be assessed whether FLAG-PA100 is capable of reducing cell viability in NSC-34 cells (Fig. 2a) and PCNs (Fig. 2c).

To examine whether the poly-PR-induced neuronal cell death is dependent on the PR repeat number, we compared the magnitude of death in cells adenovirally overexpressing EGFP-FLAG-PR5 and EGFP-FLAG-PR100 at a similar expression level (Fig. 2e, f). The expression level of EGFP-FLAG-PR5 at a multiplicity of infection (MOI) of 100, 200, or 400 was slightly higher than that of EGFP-FLAG-PR100 at an MOI of 200, 400, or 800, respectively (Fig. 2f, dot blotting analysis). We found that the expression of EGFP-FLAG-PR5 did not reduce cell viability of NSC-34 cells, whereas that of EGFP-FLAG-PR100 at MOIs of 400 and 800 significantly reduced it in an expression level-dependent manner (Fig. 2e, f). These results indicated that poly-PR induces neuronal cell death in a repeat length- and expression level-dependent manner.

### Poly-PR induces JNK-mediated apoptotic neuronal cell death

**Fig. 3:**
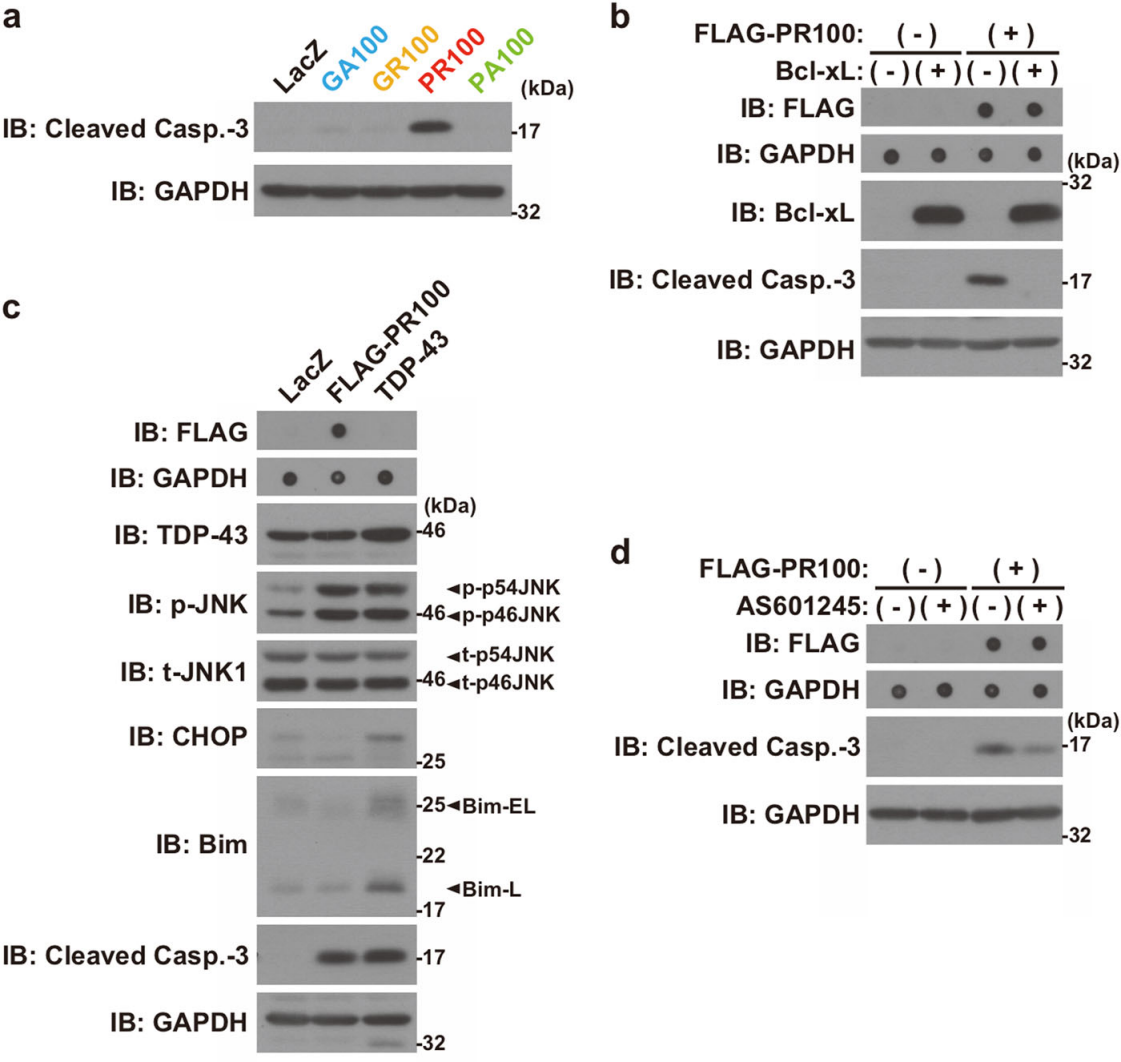
PR100 induces JNK-mediated apoptotic neuronal cell death. **a** NSC-34 cells were infected with adenovirus encoding LacZ or FLAG-DPR at an MOI of 800 together with adenovirus encoding Cre-recombinase at an MOI of 40. At 48 h after the infection, the cell lysates were subjected to immunoblotting (IB) analysis using Cleaved Caspase-3 antibody. The expression levels of each FLAG-DPR100 were shown in Fig. 2b. **b** NSC-34 cells were co-infected with adenovirus encoding LacZ (−) or FLAG-PR100 (+) at an MOI of 400 together with adenovirus encoding LacZ (−) or Bcl-xL (+) at an MOI of 200. All samples were co-infected with adenovirus encoding Cre-recombinase at an MOI of 40. At 48 h after the infection, the cell lysates were subjected to immunoblotting (IB) and dot blotting analysis using indicated antibodies. **c** NSC-34 cells were infected with adenovirus encoding LacZ, FLAG-PR100 at an MOI of 800, or TDP-43 at an MOI of 400 together with adenovirus encoding Cre-recombinase at an MOI of 40. To keep the total MOIs of adenoviruses constant, appropriate MOIs of LacZ-encoding adenovirus were added for TDP-43-infected sample. At 48 h after the infection, the cell lysates were subjected to immunoblotting (IB) and dot blotting analysis using indicated antibodies. p-JNK, phosphorylated JNK; t-JNK, total JNK. **d** NSC-34 cells were infected with adenovirus encoding LacZ (−) or FLAG-PR100 (+) at an MOI of 400 together with adenovirus encoding Cre-recombinase at an MOI of 40. After the infection, the cells were treated with (+) or without (−) 2.5 μM AS601245. At 48 h after the infection, the cell lysates were subjected to immunoblotting (IB) and dot blotting analysis using indicated antibodies

Because the expression of poly-PR caused the most prominent toxicity to neurons (Fig. 2), we focused on the analysis of the poly-PR-induced neuronal toxicity in the following study. We first found that the cleavage of caspase-3 appeared in NSC-34 cells expressing FLAG-PR100 (Fig. 3a) and it was completely inhibited by the co-expression of Bcl-xL, an apoptosis inhibitor (Fig. 3b). This result suggests that the mitochondria apoptotic pathway is involved in the poly-PR-induced neuronal cell death.

It has been reported that C9-ALS/FTD is associated with the TDP-43 pathology^6^. This clinical observation suggests that the dysregulation of TDP-43 contributes to the C9-ALS/FTD pathogenesis. Because our previous studies have shown that the TDP-43-induced apoptosis is mediated by several cell death signals involving c-Jun N-terminal kinase (JNK)/c-Jun, C/EBP homologous protein (CHOP), and Bcl-2-related BH3-only protein Bcl-2-interacting mediator of cell death (Bim)^25–27^, we examined whether the poly-PR-induced neuronal cell death is mediated with these molecules. As a consequence, we found that the poly-PR-induced death signal was associated with the phosphorylation of JNK, but not with the induction of CHOP or Bim expression (Fig. 3c). The involvement of JNK in the poly-PR-induced apoptosis was confirmed by the result indicating that the poly-PR-induced cleavage of caspase-3 was partially attenuated by the treatment of AS601245, a JNK inhibitor (Fig. 3d).

### Poly-PR reduces ribosomal RNA expression levels

**Fig. 4:**
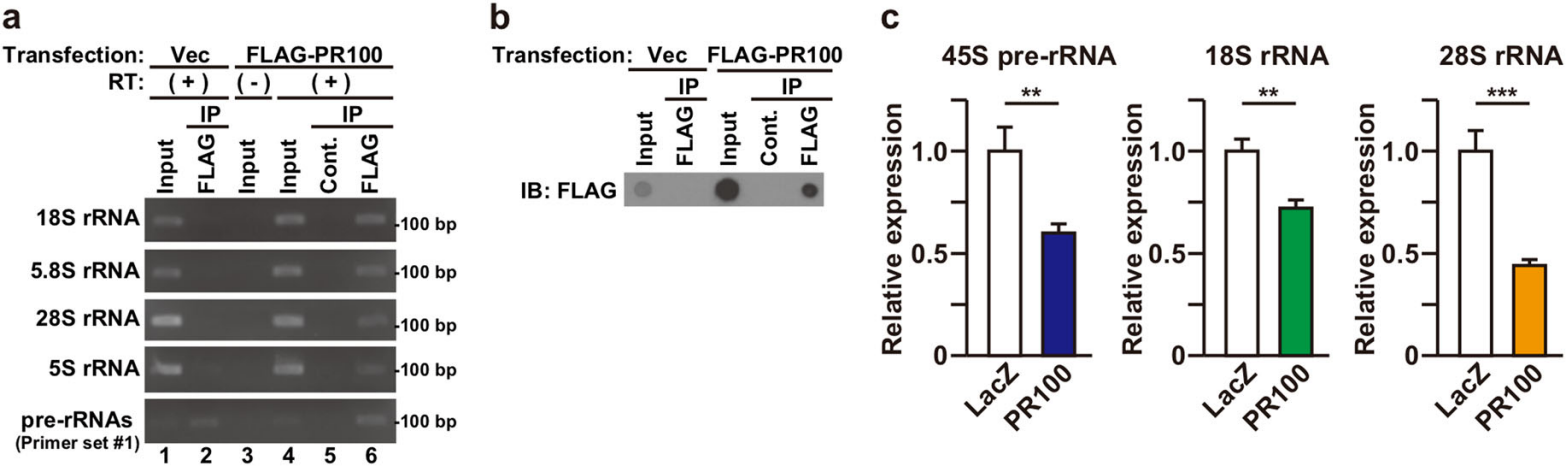
PR100 down-regulates the expression of ribosomal RNAs. **a**, **b** NSC-34 cells were transfected with the empty vector or the FLAG-PR100-encoding vector. At 48 h after the transfection, the cell lysates were immunoprecipitated (IP) with normal mouse IgG (Cont.) or FLAG antibody. Precipitates were then used for RNA immunoprecipitation (RIP) assay (**a**) and dot blotting analysis using FLAG antibody (**b**). RT (−) was used as negative control to monitor the PCR amplification from genomic DNA. Priming sites of primers are shown in Fig. S1a. **c** NSC-34 cells were infected with adenovirus encoding LacZ or FLAG-PR100 at an MOI of 800 together with adenovirus encoding Cre-recombinase at an MOI of 40. At 48 h after the infection, quantitative real time PCR analysis of 45S pre-rRNA, 18S rRNA, and 28S rRNA was performed. Means ± SD, *N* = 3 biological replicates. Statistical analysis was determined by unpaired *t* test

In the nucleolus, rRNAs, 18S, 5.8S, and 28S, are generated from a common precursor rRNAs that is transcribed by RNA polymerase I (Fig. S1a), whereas 5S rRNA is generated separately by RNA polymerase III^28,29^. The nucleolar localization of EGFP-FLAG-PR100, suggested in Fig. 1e, subsequently led us to examine the interaction between poly-PR and rRNAs, using an RNA immunoprecipitation assay. We found that the amplification of portions corresponding to 18S, 5.8S, 28S, and 5S rRNA occurred in the FLAG-PR100 immunoprecipitates, whereas that of portions corresponding to internal transcribed spacer 1 (ITS1) and 5’-external transcribed spacer (5’-ETS) of pre-rRNAs or intermediate rRNAs did not occur (Fig. 4a, b, and Fig. S1a, b). This result indicates that FLAG-PR100 binds to 5S rRNA, but not to some pre-rRNAs or intermediate rRNAs (47S, 45S, 41S, 36S, 34S, and 20S). It is also highly likely that FLAG-PR100 binds to mature 18S, 5.8S, and 28S rRNAs although it is theoretically possible that the amplification of the portions, corresponding to 18S, 5.8S, and 28S rRNA, may occur from some intermediate rRNAs such as 32S, 18S-E, 12S, and 7S rRNA (Fig. S1a). Furthermore, quantitative real-time PCR analysis indicated that FLAG-PR100 significantly down-regulated the levels of 45S pre-rRNA, 18S rRNA, and 28S rRNA expression (Fig. 4c). This result suggests that the expression of poly-PR results in the impairment of ribosome biogenesis. Impairment of rRNA biogenesis by poly-PR has also been suggested by independent groups^21,22^.

### Poly-PR-induced neuronal cell death is mediated by the inhibition of ribosome biogenesis

**Fig. 5:**
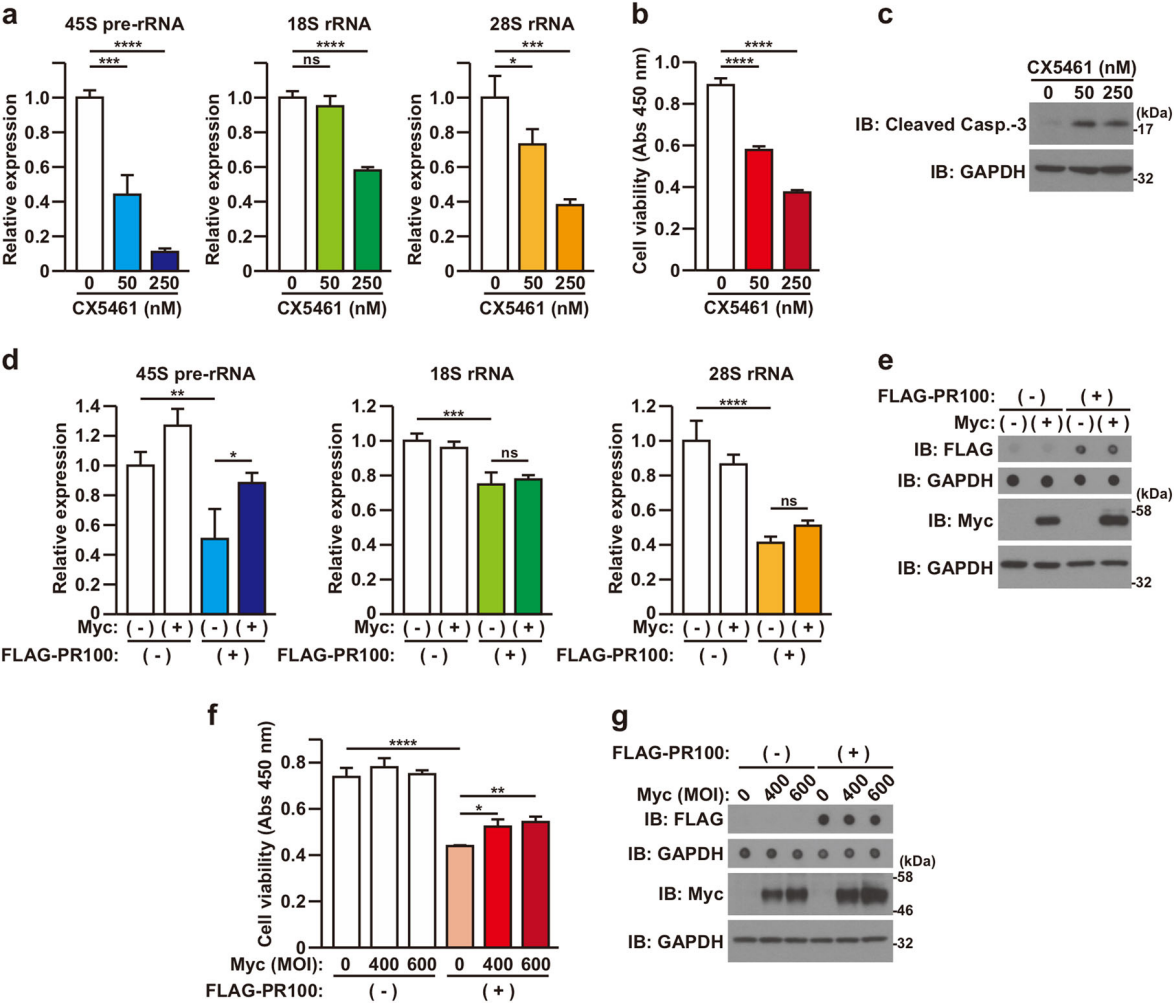
Inhibition of ribosome biogenesis mediates PR100-induced toxicity. **a**–**c** NSC-34 cells were treated with 0–250 nM CX5461. At 48 h after the treatment, quantitative real time PCR analysis of 45S pre-rRNA, 18S rRNA, and 28 S rRNA was performed (**a**). The cell viability was detected by WST-8 assay (**b**) and the cell lysates were subjected to immunoblotting (IB) analysis using Cleaved Caspase-3 antibody (**c**). Means ± SD, *N* = 3 biological replicates. Statistical analysis was determined by one-way ANOVA followed by Dunnett’s multi comparisons test. **d**, **e** NSC-34 cells were co-infected with adenovirus encoding LacZ (−) or FLAG-PR100 (+) at an MOI of 200 together with adenovirus encoding LacZ (−) or Myc (+) at an MOI of 600. All samples were co-infected with adenovirus encoding Cre-recombinase at an MOI of 40. At 48 h after the infection, quantitative real time PCR analysis of 45S pre-rRNA, 18S rRNA, and 28S rRNA was performed (**d**). The cell lysates were subjected to immunoblotting (IB) and dot blotting analysis using indicated antibodies (**e**). Means ± SD, *N* = 3 biological replicates. Statistical analysis was determined by one-way ANOVA followed by Tukey’s multi comparisons test. **f**, **g** NSC-34 cells were co-infected with adenovirus encoding LacZ (−) or FLAG-PR100 (+) at an MOI of 200 together with adenovirus encoding Myc at MOIs of 0–600. To keep the total MOIs of adenoviruses constant, appropriate MOIs of LacZ-encoding adenovirus were added for each infection. All samples were co-infected with adenovirus encoding Cre-recombinase at an MOI of 40. At 48 h after the infection, the cell viability was detected by WST-8 assay (**f**) and the cell lysates were subjected to immunoblotting (IB) and dot blotting analysis using indicated antibodies (**g**). Means ± SD, *N* = 3 biological replicates. Statistical analysis was determined by one-way ANOVA followed by Tukey’s multi comparisons test

We then investigated the involvement of ribosome biogenesis in the poly-PR-mediated neuronal toxicity. Treatment of CX5461, an RNA polymerase I inhibitor^30^, inhibited the expression of rRNAs in a dose-dependent manner (Fig. 5a). It also decreased cell viability (Fig. 5b) and induced cleavage of caspase-3 (Fig. 5c). These results raise the possibility that the inhibition of ribosome biogenesis by reducing rRNA expression contributes to neurotoxicity. It has been shown that Myc has the ability to accelerate the ribosome biogenesis by inducing the transcription of rRNA and the expression of ribosomal proteins^31–34^. Indeed, we found that the overexpression of Myc caused the level of 45S pre-rRNA expression to show an increasing tendency in NSC-34 cells (Fig. 5d, e, 45S pre-rRNA, compare lanes 1 and 2). Notably, the overexpression of Myc recovered the expression of 45S pre-rRNA that was down-regulated by poly-PR (Fig. 5d, e, compare lanes 3 and 4). Importantly, the overexpression of Myc partially restored the cell viability that was impaired by poly-PR in an expression level-dependent manner (Fig. 5f, g). These results suggest that the poly-PR-induced neuronal cell death is mediated by, at least in part, the inhibition of ribosome biogenesis, although these results don’t rule out the possibility that the expression of Myc restored poly-PR-induced neuronal toxicity by regulating other signaling pathways than the acceleration of ribosome biogenesis.

### Poly-PR interacts with multiple DEAD-box RNA helicases in an RNA-dependent manner

**Fig. 6:**
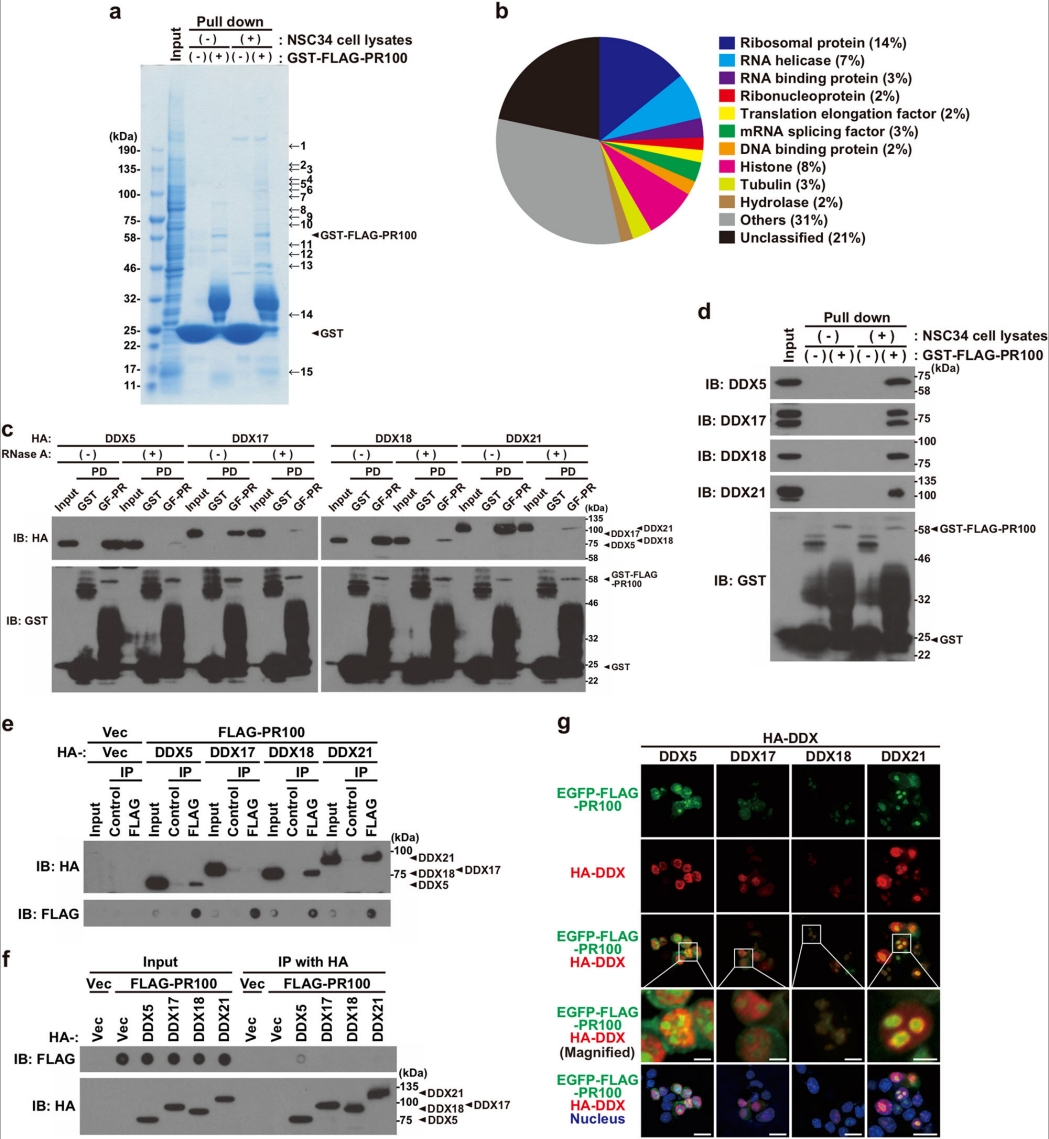
PR100 interacts with DEAD-box RNA helicases in an RNA-dependent manner. **a**, **b** Purified recombinant GST (−) or GST-FLAG-PR100 (+)-bound glutathione beads were mixed with (+) or without (−) NSC-34 cell lysates. After 18 h of rotation at 4 °C, the glutathione beads were washed, fractionated by 5-20% gradient-gel SDS-PAGE, and stained with Coomassie brilliant blue (CBB) (**a**). PR100-binding proteins (bands 1–15) were isolated and identified by mass-spectrometry analysis. Identified proteins were categorized by PANTHER Classification System (**b**). **c** NSC-34 cell lysates overexpressing HA-tagged DDX5, DDX17, DDX18, or DDX21 and purified recombinant GST or GST-FLAG-PR100 (GF-PR)-bound glutathione beads were incubated with or without 20 μg/mL RNase A. After the incubation, the cell lysates were mixed with recombinant GST or GST-FLAG-PR100-bound glutathione beads. The glutathione beads were washed and subjected to immunoblotting (IB) using indicated antibodies. The large smear within the molecular weights ranging 25–46 kDa is thought to consist of C-terminal truncated GST-FLAG-PR100 proteins. A band located around 50 kDa in the GST lane is thought to be dimerized GST and/or aggregated GST-derived proteins. PD, pull down **d** Purified recombinant GST (−) or GST-FLAG-PR100 (+)-bound glutathione beads were mixed with (+) or without (−) NSC-34 cell lysates. After 4 h of rotation at 4 °C, the glutathione beads were washed and subjected to immunoblotting (IB) using indicated antibodies. The large smear within the molecular weights ranging 25–46 kDa is thought to consist of C-terminal truncated GST-FLAG-PR100 proteins. A band located around 50 kDa in the GST lane is thought to be dimerized GST and/or aggregated GST-derived proteins. **e**, **f** NSC-34 cells, transiently transfected with indicated vectors, were harvested at 48 h after the transfection and the prepared cell lysates were subjected to immunoprecipitation (IP) with the FLAG, control (**e**), or HA (**f**) antibody. The washed precipitates were fractionated by SDS-PAGE, followed by immunoblotting (IB). The precipitates were also spotted onto PVDF membranes for dot blotting analysis. **g** NSC-34 cells overexpressing EGFP-FLAG-PR100 (green) together with HA-tagged DDX5, DDX17, DDX18, or DDX21 were fixed and immunostained with HA (red). Nuclei were stained with Hoechst33258 or DAPI (blue). Scale bar: 20 μm

To further elucidate the molecular mechanisms underlying the poly-PR-induced neuronal cell death, we searched for poly-PR-binding proteins by performing pull-down analysis of NSC-34 cell lysates using recombinant GST or GST-FLAG-PR100 as baits. After being washed, the pulled-down precipitates containing GST or GST-FLAG-PR100 and its interactors were subjected to SDS-PAGE and stained with Coomassie brilliant blue (CBB). Multiple visualized FLAG-PR100-binding proteins were isolated and identified by mass-spectrometry analysis (Fig. 6a, No. 1–15). As expected, numerous ribosomal proteins were identified as poly-PR-binding proteins (Fig. 6b). In addition, multiple proteins involving RNA metabolism including RNA helicases, ribonucleoproteins, translation elongation factors, and RNA splicing regulators were also identified (Fig. 6b). Considering that poly-PR predominantly localizes in the nucleolus (Fig. 1e), we initially focused on nucleolus-localizing proteins (Table S1). In particular, we investigated the role of some DEAD-box RNA helicases (DDXs) including DEAD-box RNA helicase 5 (DDX5), DDX17, DDX18, and DDX21 in the poly-PR-induced neuronal cell death because it has been known that several DDXs are involved in the regulation of ribosome biogenesis^16^. Using another GST pull-down assays, we confirmed that not only exogenously overexpressed HA-tagged DDX5, DDX17, DDX18, and DDX21 (Fig. 6c), but also endogenously expressed DDX5, DDX17, DDX18, and DDX21 (Fig. 6d), bound to GST-FLAG-PR100. Consistently, co-immunoprecipitation assays confirmed that HA-tagged DDX5, DDX18, and DDX21 were co-immunoprecipitated with FLAG-PR100 (Fig. 6e). Reciprocally, FLAG-PR100 was co-immunoprecipitated with HA-tagged DDX5 (Fig. 6f). However, the interaction between FLAG-PR100 and HA-DDX17 was not observed by the co-immunoprecipitation assay, probably because the FLAG or the HA antibody are incapable of precipitating the FLAG-PR100 complexed with HA-DDX17 or HA-DDX17 complexed with FLAG-PR100, respectively. Given that DDXs are RNA-binding proteins and poly-PR binds to rRNAs (Fig. 4a, b, Fig. S1b), it is assumed that the interaction between poly-PR and DDXs is dependent on the presence of RNA. In agreement, we found that the treatment with RNase A markedly reduced their interactions (Fig. 6c). This result suggests that the interaction between poly-PR and DDXs is mediated by RNA. Furthermore, immunocytochemical analysis showed that DDX18 partially co-localized with EGFP-FLAG-PR100 in the nucleolus (Fig. 6g). The other DDXs, DDX5, DDX17, and DDX21, formed ring-like structure around and outside of the poly-PR aggregates and -distributing area (Fig. 6g). These results together suggest that these DDXs functionally interact with poly-PR.

### Reduction in the expression of some DEAD-box RNA helicases down-regulates rRNA levels and causes neuronal cell death

**Fig. 7:**
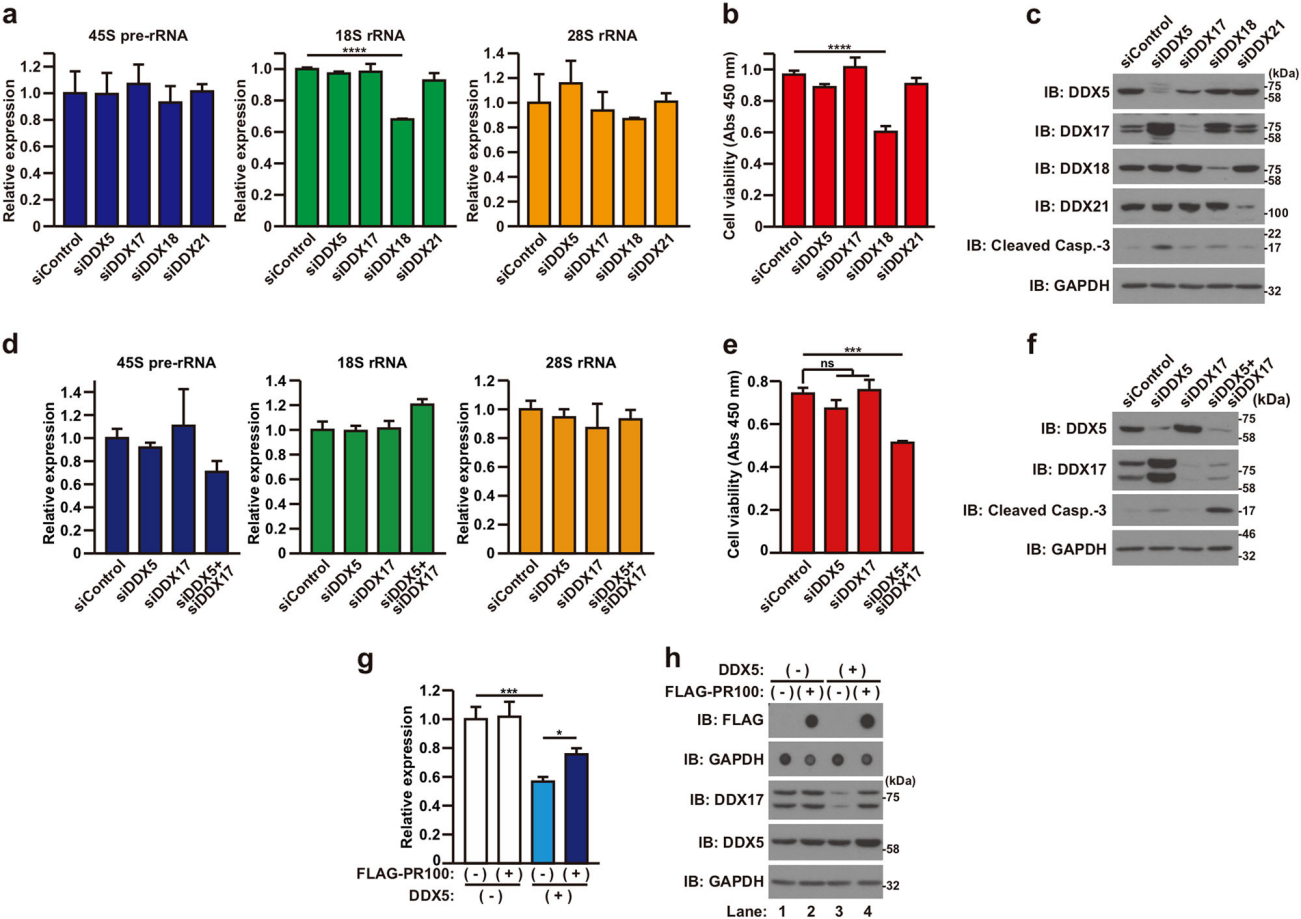
Knockdown of DDXs reduces rRNA expression and cell viability. **a**–**c** NSC-34 cells were transfected with 5 nM control, DDX5-#1, DDX17, DDX18, or DDX21 siRNA. At 60 h after the transfection, quantitative real time PCR analysis of 45S pre-rRNA, 18S rRNA, and 28S rRNA was performed (**a**). The cell viability was detected by WST-8 assay (**b**) and the cell lysates were subjected to immunoblotting (IB) analysis using indicated antibodies (**c**). Means ± SD, *N* = 3 biological replicates. Statistical analysis was determined by one-way ANOVA followed by Dunnett’s multi comparisons test. **d**–**f** NSC-34 cells were co-transfected with 5 nM control, DDX5-#2, and DDX17 siRNA as indicated. To keep the total concentration of siRNA constant at 10 nM, appropriate amount of control siRNA was added for each transfection. At 60 h after the transfection, quantitative real time PCR analysis of 45S pre-rRNA, 18S rRNA, and 28S rRNA was performed (**d**). The cell viability was detected by WST-8 assay (**e**) and the cell lysates were subjected to immunoblotting (IB) analysis using indicated antibodies (**f**). Means ± SD, *N* = 3 biological replicates. Statistical analysis was determined by one-way ANOVA followed by Dunnett’s multi comparisons test. **g**, **h** NSC-34 cells were co-infected with adenovirus encoding LacZ (−) or FLAG-PR100 (+) at an MOI of 400 together with adenovirus encoding LacZ (−) or DDX5 (+) at an MOI of 400. All samples were co-infected with adenovirus encoding Cre-recombinase at an MOI of 40. At 48 h after the infection, quantitative real time PCR analysis of DDX17 was performed (**g**). The cell lysates were subjected to immunoblotting (IB) and dot blotting analysis using indicated antibodies (**h**). Means ± SD, *N* = 3 biological replicates. Statistical analysis was determined by one-way ANOVA followed by Tukey’s multi comparisons test

DDXs are positively involved in ribosome biogenesis^16,28^. It could be therefore hypothesized that poly-PR down-regulates ribosome biogenesis by inhibiting the function of DDXs (the loss-of-function of DDXs). Based on this hypothesis, we first examined the effect of siRNA-mediated silencing of endogenous DDXs expression on rRNA expression and cell viability. Knockdown of DDX18 significantly decreased the expression of 18S rRNA (Fig. 7a, c) as well as the cell viability (Fig. 7b) and slightly induced the cleavage of caspase-3 (Fig. 7c). Knockdown of DDX5 did not affect rRNA expression (Fig. 7a) or the cell viability (Fig. 7b) but induced the cleavage of caspase-3 (Fig. 7c). Neither the knockdown of DDX17 nor the knockdown of DDX21 affected the expression of rRNA or the cell viability (Fig. 7a–c). It has been previously shown that DDX5 and DDX17 have redundant roles in ribosome biogenesis and cell growth^35,36^. Therefore, we assumed that the reduction in both DDX5 and DDX17 expression may affect rRNA expression and cell viability. Knockdown of both DDX5 and DDX17 expression tended to reduce 45S pre-rRNA expression (Fig. 7d, 45S pre-rRNA, unpaired t test *p* < 0.05) that remained insignificant by multiple comparisons test (*p* = 0.16). As expected, it decreased the cell viability (Fig. 7e) and induced the cleavage of caspase-3 synergistically (Fig. 7f, compare lanes 2, 3, and 4). Given that poly-PR down-regulates all of 45S pre-rRNA, 18S rRNA, and 28S rRNA (Fig. 4c), these data suggest that the loss-of-function of DDX5/DDX17 or DDX18 mimics some part of the poly-PR-induced down-regulation of rRNA levels that leads to neuronal cell death.

### Poly-PR inhibits the function of DDX5

We next asked whether poly-PR inhibits the function of DDXs. It has been demonstrated that DDX5 down-regulates the expression of DDX17^35,36^. Consistent with the previous studies^35,36^, it was observed that the knockdown of DDX5 significantly up-regulates the level of DDX17 expression (Fig. 7c, f, compare lanes 1 and 2) and that the overexpression of DDX5 down-regulates the level of DDX17 at both RNA and protein levels (Fig. 7g, h, compare lanes 1 and 3). The level of exogenous DDX5 overexpression was relatively small, as compared to the endogenous expression level, possibly because of negative autoregulation of DDX5 expression^35,36^. Employing this mutual expression-regulating mechanism, we examined whether poly-PR affects the activity of DDX5 by monitoring the DDX5-mediated down-regulation of DDX17 expression. If poly-PR inhibits the activity of DDX5, it is expected that poly-PR overexpression attenuates the DDX5-mediated down-regulation of DDX17 expression. As expected, we found that poly-PR attenuated the DDX5-mediated down-regulation of DDX17 expression (Fig. 7g, h, compare lanes 3 and 4). It was confirmed that poly-PR did not affect the expression level of DDX17 (Fig. 7g, h, compare lanes 1 and 2). These results suggest that poly-PR inhibits the function of DDX5.

## Discussion

Although it has been shown that multiple DPR proteins are expressed in the central nervous systems of patients with C9-ALS/FTD^13^, the relative contribution of each DPR protein to the pathogenesis have not been fully elucidated. In the current study, the results of the cell death assay (Fig. 2) showed that poly-PR had the strongest neurotoxicity. Poly-GR may be another potent cell-death inducer if expression levels of DPR proteins are adjusted (Fig. 2), as shown in earlier reports^37,38^. These findings suggest that arginine-containing DPR proteins are generally toxic. On the other hand, poly-GA is slightly toxic in both NSC-34 cells and primary neurons (Fig. 2). This result is consistent with the result of previous studies indicating that arginine-containing DPR proteins show stronger toxicity than poly-GA^24,37,39^.

Poly-PR predominantly localizes in the nucleolus and reduces the expression of rRNA (Figs. 1e and 4c). The co-localization of poly-PR and nucleophosmin, a nucleolar protein, has been already shown in C9-ALS spinal cord^24^. It has also been shown that the treatment with poly-PR20 alters rRNA processing in astrocytes^21^, that the expression of GFP-tagged poly-PR30 decreases 18S and 28S rRNA levels in HEK293 cells^22^, and that 45S pre-rRNA maturation is decreased in patients with C9ORF72 mutation^40^. Furthermore, it has recently been reported that poly-PR reduces the expression of several ribosomal proteins and co-aggregates with them in the brains of patients with C9ORF72 mutation^41^. In the current study, we have shown that the reduction in the expression of rRNA, caused by an RNA polymerase I inhibitor, results in neuronal cell death (Fig. 5a–c) and that the increase in the expression of rRNA by the overexpression of Myc, an accelerator of ribosome biogenesis^31–34^, counteracts the poly-PR-induced neuronal cell death (Fig. 5d–g). These findings together suggest that poly-PR causes neuronal cell death at least partially by reducing rRNA levels that lead to the inhibition of ribosome biogenesis.

The overexpression of Myc partially restored the cell viability that was impaired by poly-PR (Fig. 5f, g). We have concluded that the poly-PR-induced neuronal cell death is mediated by, at least in part, the inhibition of ribosome biogenesis. It is also possible that the expression of Myc restored the poly-PR-induced neuronal toxicity in a ribosome biogenesis-independent mechanism.

The overexpression of Myc had no or mild effect on the expression levels of 18S or 28S rRNA that were down-regulated by poly-PR in the employed assay condition (Fig. 5d). However, it clearly recovered the level of 45S pre-rRNA that was down-regulated by poly-PR (Fig. 5d). The main reason for this discrepancy may be that the expression levels of 18S or 28S rRNA are less sensitively affected by the activation of RNA polymerase I than that of the 45S pre-rRNA level in the short-term assay condition employed. Consistently with this explanation, the decrease in the level of 45S pre-rRNA that is induced by CX5461, a selective chemical inhibitor of RNA polymerase I, is also larger than those of 18S and 28S rRNA, as shown in Fig. 5a. Importantly, because the 45S pre-rRNA is the precursor of 18S and 28S rRNA, it is natural to think that the levels of 18S and 28S rRNA show a significant tendency to increase after the 45S pre-rRNA level is increased.

Given that poly-PR reduces the expression of several ribosomal proteins^41^ and that Myc up-regulates transcription of many ribosomal proteins and co-factors that are required for ribosome maturation^34,42–44^, it is likely that the overexpression of Myc also recovers the expression of ribosomal proteins and co-factors that are down-regulated by poly-PR. In addition, Myc also activates RNA polymerase III, leading to increase of expression of 5S rRNA, which are transcribed by RNA polymerase III in the nucleus^45,46^. However, in the current study, the lack of probes and primers that can exactly and strictly quantify the levels of short length rRNA such as 5.8S rRNA and 5S rRNA leave us unable to show these rRNA alterations.

The inhibition of ribosome biogenesis by poly-PR may be mediated by multiple concurrently progressing mechanisms. In the current study, we identified DEAD-box RNA helicases as targets of poly-PR to impair the ribosome biogenesis. In this relation, it has been previously shown that DEAD-box RNA helicases positively regulate ribosome biogenesis^16,28^ and that the reduction in the expression of DEAD-box RNA helicases including DDX5, DDX17, DDX18, and DDX21 decreases ribosome biogenesis^35,47–50^. In the current study, we showed that the knockdown of DDX5/DDX17 or DDX18 causes the reduction of 45S pre-rRNA level or 18S rRNA, respectively, in parallel to the decrease in cell viability (Fig. 7). On the other hand, knockdown of DDX5/DDX17, DDX18, or DDX21 did not affect the expression levels of 28S rRNA (Fig. 7). Consequently, the data provided in the current study do not explain the full characteristics of the poly-PR-mediated down-regulation of rRNAs including 28S rRNA (Fig. 4c). There are numerous DEAD-box RNA helicases other than DDX5, DDX17, DDX18 and DDX21^14^. Therefore, it is likely that poly-PR also interacts with and inhibits the function of some undetermined DEAD-box RNA helicases. Furthermore, it is also possible that poly-PR interacts with and inhibits the function of some undetermined other-type of RNA helicases. The summation of the poly-PR-mediated inhibition of the function of such undetermined RNA helicases as well as DDX5/DDX17 and DDX18 may explain the full characteristics of the poly-PR-mediated down-regulation of rRNAs.

Regarding the poly-PR-mediated inhibition of the function of DDXs, we have provided evidence indicating that poly-PR inhibits the function of DDX5 (Fig. 7g, h). Because the detailed functions of most DDXs remain undetermined and, as a result, assays to test their function are unavailable currently, we could not address to the question as to whether poly-PR inhibits the function of the other DDXs. However, given that poly-PR binds to DDX17, 18, and 21, it is highly likely that it inhibits the function of these DDXs similarly.

Thus, the current study implicated DEAD-box RNA helicases as mediators of the poly-PR-mediated inhibition of ribosome biogenesis and neurotoxicity. However, this class of molecules may not be the only mediators of the poly-PR-mediated inhibition of ribosome biogenesis and neurotoxicity. Given a number of ribosomal proteins were identified as poly-PR interactors (Fig. 6a, b), it is possible that poly-PR may interact with and affect the function of ribosomal proteins that positively or negatively regulate ribosome biogenesis. Consistently, it has recently been reported that poly-PR reduces the expression of several ribosomal proteins^41^. It is also likely that poly-PR directly regulates rRNA expression through the inhibition of transcription and/or the promotion of instability of rRNAs. In fact, we have found that poly-PR directly or indirectly binds to rRNA species (Fig. 4a, b, Fig. S1b).

In summary, we have shown that poly-PR causes neuronal cell death by binding to DEAD-box RNA helicases and inhibiting the functions of them that leads to the inhibition of ribosome biogenesis. The results provide some insight into the mechanism underlying the C9-ALS/FTD pathogenesis. Further investigations that include identification of mediators involved in the inhibition of ribosome biogenesis beside DEAD-box RNA helicases and pathophysiological relevance of ribosome dysfunction in vivo need to be carried out to fully clarify the mechanism underlying C9ORF72-linked neurodegeneration.

## Materials and methods

### Antibodies and compounds

The following antibodies were purchased from suppliers: Bcl-xL (#sc-634), CHOP (#sc-793), DDX5 (#sc-365164), DDX17 (#sc-398168), DDX21 (#sc-376758), GST (#sc-138), and JNK1 (#sc-571), Santa Cruz Biotechnology (Dallas, TX, USA); Bim (#2933), Cleaved Caspase-3 (#9661), Fibrillarin (#2639), GAPDH (#2118), and phospho-JNK (#9255), Cell Signaling Technology (Danvers, MA, USA); FLAG (#F1804) and horseradish peroxidase (HRP)-conjugated FLAG (#A8592), Sigma-Aldrich (St. Louis, MO, USA); TDP-43 (#12892-1-AP), Proteintech Group (Rosemont, IL, USA); HA (#11867423001) and HRP-conjugated HA (#12013819001), Roche Diagnostics (Basel, Swiss); DDX18 (#A300-636A), Bethyl Laboratories (Montgomery, TX, USA); Myc (#BML-SA294-0500), Enzo Life Sciences (Farmingdale, NY, USA). AS601245 and CX5461 were purchased from Merck Millipore (Burlington, MA, USA) and AdooQ BioScience (Irvine, CA, USA), respectively. RNase A were purchased from Wako (Osaka, Japan).

### Plasmids

cDNAs encoding FLAG-tagged GA100, GR100, PR100, PA100, or PR5 were synthesized (Thermo Fisher Scientific, Waltham, MA, USA) and subcloned into pEF1/myc-His vector (Thermo Fisher Scientific) or pEGFP-C1 vector (Takara, Shiga, Japan) to express FLAG-tagged or EGFP-FLAG-tagged DPR proteins, respectively. A stop codon was inserted just after the DPR protein-encoding sequence. Human DDX17 and DDX18 cDNAs were amplified from human adult normal testis cDNA (BioChain Institute, Newark, CA, USA). Human DDX21 cDNA were amplified from HeLa cell cDNA. These DDX-encoding cDNAs were subcloned into pEF4/His vector (Thermo Fisher Scientific) in which HA tag-encoding sequence was inserted before Xpress tag-encoding sequence to express HA-tagged DDX17, DDX18, and DDX21. Human DDX5-HA-myc-His_6_-encoding plasmid was kindly provided by Dr. Didier Auboeuf (Université de Lyon)^51^. Human Myc cDNA was amplified from human adult normal testis cDNA (BioChain Institute).

### Adenovirus vectors

The adenovirus expression vector systems were purchased from Takara. Bcl-xL, Cre-recombinase, LacZ, and TDP-43-encoding adenovirus vectors were described previously^25^. The cDNAs encoding FLAG-tagged GA100, GR100, PR100, PA100, EGFP-FLAG-tagged PR5, EGFP-FLAG-tagged PR100, Myc, and DDX5 were inserted into the *Swa*I site of a cosmid adenoviral vector, pAxCALNLw. In this vector, a stuffer DNA fragment, sandwiched between two loxP sequences, is located just upstream of cDNA and interferes with gene expression. If an adenovirus vector expressing Cre-recombinase is co-introduced into the cells, the stuffer is removed, and the gene is expressed. All viruses were grown in HEK293 cells and purified using CsCl gradient ultracentrifugation. NSC-34 cells, seeded at 1 × 10^5^ cells per well on 6-well plates or 5 × 10^4^ cells per well on 12-well plates, were incubated with media containing adenovirus vector at the indicated multiplicity of infection (MOI) at 37 °C.

### Cell culture and transfection

NSC-34 motor neuron cell, a hybrid cell line established from a mouse neuroblastoma cell line and mouse embryo spinal cord cells, was a kind gift from Dr. Neil Cashman (University of Toronto). HEK293 cells were derived from our laboratory depository. The absence of mycoplasma contamination of these cell lines was validated using MycoAlert™ Mycoplasma Detection Kit (Takara). NSC-34 cells and HEK293 cells were grown in Dulbecco’s modified Eagle’s medium (DMEM) (Wako), supplemented with 10% of fetal bovine serum (FBS) (GE Healthcare UK Ltd, Buckinghamshire, England) and antibiotics (Thermo Fisher Scientific). Transfection was performed using Lipofectamine (Thermo Fisher Scientific) and PLUS reagent (Thermo Fisher Scientific) or Lipofectamine 2000 (Thermo Fisher Scientific) under the manufacturer’s protocol.

### Primary neurons

Primary cultured cerebral cortical neurons (PCNs), obtained from embryonic day 14 ICR mice, were seeded on poly-l-lysine-coated 96-well plates (Sumitomo Bakelite, Tokyo, Japan) at 5 × 10^4^ cells/well or poly-l-lysine-coated 6-well plates (Sumitomo Bakelite) at 1 × 10^6^ cells/well in Neuron medium (Sumitomo Bakelite). Purity of neurons by this method was >98%. PCNs were infected with adenoviruses at the indicated MOI in Neuron medium at 37 °C.

### Western blotting and dot blotting analysis

Cells were lysed in a cell lysis buffer [10 mM Tris-HCl (pH 7.4), 1% Triton X-100, 1 mM EDTA, protease inhibitors] by a freeze-thaw cycle or in a 4% SDS-containing sample buffer by sonication. Protein concentration was measured by Pierce BCA Protein Assay Kit (Thermo Fisher Scientific). The samples in the SDS-containing sample buffer were boiled for 5 min at 95 °C, fractionated by SDS–PAGE, and blotted onto polyvinylidene fluoride (PVDF) membranes (Pall Corporation, Port Washington, NY, USA). For dot blotting analysis, 1 μL of cell lysates in a 4% SDS-containing sample buffer was spotted onto PVDF membranes. After blocking with 5% skim milk (Becton, Dickinson and Company, Sparks, MD, USA) in TBST, immunoblotting was performed with indicated antibodies. Immunoreactive bands were detected with ECL Western Blotting Detection Reagents (GE Healthcare UK Ltd). Glyceraldehyde 3-phosphate dehydrogenase (GAPDH) was used as an internal control.

### Cell viability assay

Cell viability was measured by WST-8 cell viability assays. The WST-8 assay, performed using Cell Counting Kit-8 (Dojindo, Osaka, Japan), was based on the ability of cells to convert a water-soluble WST-8 into a water-soluble WST-8 formazan. Cells were treated with WST-8 reagent at 37 °C for 1–5 h, and 450 nm absorbance was measured.

### Immunocytochemistry

NSC-34 cells were transfected using Lipofectamine 2000 (Thermo Fisher Scientific) under the manufacturer’s protocol. At 48 h after the transfection, the cells were fixed with 4% paraformaldehyde-PBS and immunostained using the Fibrillarin antibody or HA antibody and the secondary antibody, Cy3-labeled goat anti-rabbit IgG antibody (Jackson ImmunoResearch Inc., West Grove, PA, USA) or Alexa Fluor 568-labeled goat anti-rat IgG antibody (Thermo Fisher Scientific). Nuclei were stained with Hoechst 33258 (Sigma-Aldrich) or Hard Set Mounting Medium with DAPI (Vector Laboratories, Inc., Burlingame, CA, USA). The cells were observed using a confocal microscope LSM710 with ZEN 2010 software (Carl Zeiss, Oberkochen, Germany).

### Quantitative real-time PCR analysis

Total RNA was extracted from NSC-34 cells treated with CX5461, infected with indicated adenovirus vectors, or transfected with indicated siRNAs, using RNeasy Plus Mini kit (QIAGEN, Hilden, Germany). Genomic DNA was minimized with gDNA eliminator spin columns and DNase treatment using RNase-Free DNase Set (QIAGEN). Reverse transcription and PCR reactions were performed on an Applied Biosystems StepOnePlus^TM^ Real-Time PCR System (Thermo Fisher Scientific) using the Taqman RNA-to-Ct 1-Step Kit (Thermo Fisher Scientific). The pairs of primers and the Taqman probes for target RNAs were designed based on mouse RNA sequences using Taqman Gene Expression Assays (Thermo Fisher Scientific). Assay IDs of Taqman probe for mouse 45S pre-rRNA, 18S rRNA, 28S rRNA, Ddx17, and Gapdh are Mm03985792_s1, Mm03928990_g1, Mm03682676_s1, Mm01300135_m1, and Mm99999915_g1, respectively. Data analysis was performed using StepOne Software ver. 2.0.2 (Thermo Fisher Scientific). Relative expression was analyzed by the relative standard curve method. Data were normalized to the mRNA expression of GAPDH.

### RNA immunoprecipitation (RIP) assay

RIP assay was performed using Magna RIP RNA-Binding Protein Immunoprecipitation Kit (Merck Millipore) under the manufacturer’s protocol. Briefly, NSC-34 cells, transfected with the empty vector or the FLAG-PR100-encoding vector, were lysed in a complete RIP lysis buffer. After centrifugation at 12,000*g* for 10 min, the cell lysates were incubated with normal mouse IgG_1_ (Santa Cruz Biotechnology) or anti-FLAG antibody (Sigma-Aldrich)-bound Magnetic beads overnight at 4 °C by rotation. After washing six times using cold RIP wash buffer, RNA was extracted from precipitates. First-strand cDNAs were synthesized from purified RNA using QuantiTect Rev. Transcription Kit (QIAGEN). PCR amplification with KOD-Plus-Ver.2 (TOYOBO, Osaka, Japan) was performed under denaturation at 98 °C for 10 s, annealing at 60 °C for 30 s, and elongation at 68 °C for 30 s, repeated by 19–27 cycles. The sequences of forward and reverse primers are as follows (Fig. S1a): mouse 45S pre-rRNA (Primer set-#1), sense: 5′-GTACCTAGCTGTCGCGTTCC-3′, antisense: 5′-CATGGAGTCTGAGGGAGAGC-3′; mouse 45S pre-rRNA (Primer set-#2), sense: 5′-CTCCTAGGTGCCTGCTTCTG-3′, antisense: 5′-CTCTCACGGGCTTCTCAGAC-3′; mouse 18S rRNA (Primer set-#3), sense: 5′-CCTGCGGCTTAATTTGACTC-3′, antisense: 5′-AGACAAATCGCTCCACCAAC-3′; mouse 5.8S rRNA (Primer set-#4), sense: 5′-GACTCTTAGCGGTGGATCACTC-3′, antisense: 5′-AAGTGCGTTCGAAGTGTCG-3′; mouse 28S rRNA (Primer set-#5), sense: 5′-AGTAACGGCGAGTGAACAGG-3′, antisense: 5′-GCCTCGATCAGAAGGACTTG-3′; mouse 5S rRNA, sense: 5′-GCCATACCACCCTGAACG-3′, antisense: 5′-GCCTACAGCACCCGGTATTC-3′. PCR amplicons were validated by sequence analysis. Five percent of final IP sample were used as input. In parallel with RIP assay, a part of input and IP samples was subjected to dot blotting analysis.

### Identification of poly-PR-binding proteins

Bacteria-derived recombinant GST-fused FLAG-PR100 was prepared as a bait. NSC-34 cell lysates, solubilized in lysis buffer [150 mM NaCl, 20 mM HEPES (pH 7.4), 1 mM EDTA, 1 mM DTT, 0.1% Triton X-100, protease inhibitors] by sonication and pre-cleared with glutathione beads (GE Healthcare UK Ltd), were mixed with recombinant GST or GST-FLAG-PR100-bound glutathione beads overnight at 4 °C by rotation. After washing five times using the lysis buffer, the precipitates were fractionated by 5–20% gradient gel (Wako) SDS-PAGE and stained with CBB (Sigma-Aldrich). The CBB-stained protein bands were carefully excised from the gel, washed and destained with acetonitrile (ACN). After being reduced with 10 mM DTT, proteins were alkylated with 55 mM iodoacetoamide and digested for 16 h at 37 °C with sequence-grade trypsin (Promega, Madison, WI, USA). The resulting peptides were sequentially extracted from the gel with 0.1% trifluoroacetic acid (TFA)/2% ACN, 0.1% TFA/33% ACN, and 0.1% TFA/70% ACN. The combined solutions were evaporated, and the peptides were analyzed by a nano-LC-ESI-MS/MS system consisting of DiNa nano-LC (KYA Technologies, Tokyo, Japan) and QSTAR Elite hybrid liquid chromatography tandem mass spectrometry (LC/MS/MS) (Thermo Fisher Scientific). Proteins were identified using ProteinPilot software version 3.0 (Thermo Fisher Scientific) with default parameters using the UniprotKB database (mouse).

### Pull-down assay

NSC-34 cells, transiently transfected with indicated vectors, were harvested at 48 h after the transfection and lysed in a pull-down buffer [150 mM NaCl, 20 mM HEPES (pH 7.4), 1 mM EDTA, 1 mM DTT, 0.1% Triton X-100, protease inhibitors] by sonication. After centrifugation at 12,000*g* for 15 min, the cell lysates were pre-cleared with recombinant GST-bound glutathione beads for 1.5 h and the cleared supernatants were incubated with or without 20 μg/mL RNase A at room temperature for 2 h. Recombinant GST or GST-FLAG-PR100-bound glutathione beads were also incubated with or without 20 μg/mL RNase A at room temperature for 2 h in a pull-down buffer. Then, the cell lysates were incubated with recombinant GST or GST-FLAG-PR100-bound glutathione beads at 4 °C overnight by rotation. After washing four times using the pull-down buffer, the precipitates were fractionated by SDS-PAGE, followed by immunoblotting analysis.

### Immunoprecipitation

NSC-34 cells, transiently transfected with indicated vectors, were harvested at 48 h after the transfection and lysed in a lysis buffer [150 mM NaCl, 20 mM HEPES (pH 7.4), 1 mM EDTA, 1 mM dithiothreitol (DTT), 0.1% Triton X-100, protease inhibitors] by sonication. After centrifugation at 12,000*g* for 15 min, the cell lysates were pre-cleared with protein G-Sepharose (Amersham Biosciences, Pis- cataway, NJ, USA) for 2 h or overnight. The cleared supernatants were then incubated with FLAG or HA antibody for 2–3 h and precipitated with protein G-Sepharose at 4 °C for 3 h. After washing four times with the lysis buffer, the precipitates were fractionated by SDS–PAGE, followed by immunoblotting. The precipitates were also spotted onto PVDF membranes for dot blotting analysis.

### siRNA-mediated silencing

siRNAs against mouse DDX5, DDX17, DDX18, DDX21, and non-targeting control siRNA were purchased from RNAi Co., Ltd. (Tokyo, Japan). The siRNA sequence for mouse DDX5-#1 and -#2 are 5′-GCACUUUUUUCGCUAUUUAAG-3′ and 5′-GCUUCGGGAAGCUAAUCAAGC-3′, respectively. The siRNA sequence for mouse DDX17, DDX18, and DDX21 are 5′-GCAGGUGGCUGACGAUUAUGG-3′, 5′-GCAUACAAAUCCUACAUAAGA-3′, and 5′-CCUAUCUUGGUGUGUCGAUCU-3′, respectively. NSC-34 cells were transfected using Lipofectamine 2000 (Thermo Fisher Scientific) according to the manufacturer’s reverse transfection protocol. Briefly, 6 × 10^4^ cells per well on 6-well plates or 3 × 10^4^ cells per well on 12-well plates were combined with the 5 nM siRNA per each siRNA and Lipofectamine 2000 reagent complexes.

### Statistics analysis

All values in the figures are shown as means ± SD. All experiments that were statistically analyzed were performed with *N* = 3 biological replicates. Statistical analysis was performed with a one-way ANOVA, followed by post-hoc test (Dunnett’s or Tukey’s multiple comparisons test) or Student’s *t*-test using Prism 7 software (Ver. 7.0d). *p < 0.05; **p < 0.01; ***p < 0.001; ****p < 0.0001. ns, not significant.

## Electronic supplementary material

Fig. S1 and Table S1

## References

[1] Ling SC, Polymenidou M, Cleveland DW (2013). Converging mechanisms in ALS and FTD: disrupted RNA and protein homeostasis. Neuron.

[2] Taylor JP, Brown RH, Cleveland DW (2016). Decoding ALS: from genes to mechanism. Nature.

[3] Rademakers R, Neumann M, Mackenzie IR (2012). Advances in understanding the molecular basis of frontotemporal dementia. Nat. Rev. Neurol..

[4] Pottier C, Ravenscroft TA, Sanchez-Contreras M, Rademakers R (2016). Genetics of FTLD: overview and what else we can expect from genetic studies. J. Neurochem..

[5] Gao FB, Almeida S, Lopez-Gonzalez R (2017). Dysregulated molecular pathways in amyotrophic lateral sclerosis-frontotemporal dementia spectrum disorder. EMBO J..

[6] DeJesus-Hernandez M (2011). Expanded GGGGCC hexanucleotide repeat in noncoding region of C9ORF72 causes chromosome 9p-linked FTD and ALS. Neuron.

[7] Renton AE (2011). A hexanucleotide repeat expansion in C9ORF72 is the cause of chromosome 9p21-linked ALS-FTD. Neuron.

[8] Haeusler AR, Donnelly CJ, Rothstein JD (2016). The expanding biology of the C9orf72 nucleotide repeat expansion in neurodegenerative disease. Nat. Rev. Neurosci..

[9] Ash PE (2013). Unconventional translation of C9ORF72 GGGGCC expansion generates insoluble polypeptides specific to c9FTD/ALS. Neuron.

[10] Gendron TF (2013). Antisense transcripts of the expanded C9ORF72 hexanucleotide repeat form nuclear RNA foci and undergo repeat-associated non-ATG translation in c9FTD/ALS. Acta Neuropathol..

[11] Mori K (2013). Bidirectional transcripts of the expanded C9orf72hexanucleotide repeat are translated into aggregating dipeptide repeat proteins. Acta Neuropathol..

[12] Mori, K. et al. The C9orf72 GGGGCC repeat is translated into aggregating dipeptide-repeat proteins in FTLD/ALS. *Science*1335–1338 (2013). 10.1126/science.1232927 23393093

[13] Freibaum BD, Taylor JP (2017). The role of dipeptide repeats in C9ORF72-related ALS-FTD. Front Mol. Neurosci..

[14] Linder P, Jankowsky E (2011). From unwinding to clamping—the DEAD box RNA helicase family. Nat. Rev. Mol. Cell Biol..

[15] Bourgeois CF, Mortreux F, Auboeuf D (2016). The multiple functions of RNA helicases as drivers and regulators of gene expression. Nat. Rev. Mol. Cell Biol..

[16] Martin R, Straub AU, Doebele C, Bohnsack MT (2013). DExD/H-box RNA helicases in ribosome biogenesis. RNA Biol..

[17] Bates GJ (2005). The DEAD box protein p68: a novel transcriptional coactivator of the p53 tumour suppressor. EMBO J..

[18] Valdez BC (2000). Structural domains involved in the RNA folding activity of RNA helicase II/Gu protein. Eur. J. Biochem..

[19] Steimer L, Klostermeier D (2012). RNA helicases in infection and disease. RNA Biol..

[20] Chen YZ (2004). DNA/RNA helicase gene mutations in a form of juvenile amyotrophic lateral sclerosis (ALS4). Am. J. Hum. Genet..

[21] Kwon I (2014). Poly-dipeptides encoded by the C9orf72 repeats bind nucleoli, impede RNA biogenesis, and kill cells. Science.

[22] Tao Z (2015). Nucleolar stress and impaired stress granule formation contribute to C9orf72 RAN translation-induced cytotoxicity. Hum. Mol. Genet..

[23] Yamakawa M (2015). Characterization of the dipeptide repeat protein in the molecular pathogenesis of c9FTD/ALS. Hum. Mol. Genet..

[24] Wen X (2014). Antisense proline–arginine RAN dipeptides linked to C9ORF72-ALS/FTD form toxic nuclear aggregates that initiate in vitro and in vivo neuronal death. Neuron.

[25] Suzuki H, Lee K, Matsuoka M (2011). TDP-43-induced death is associated with altered regulation of BIM and Bcl-xL and attenuated by caspase-mediated TDP-43 cleavage. J. Biol. Chem..

[26] Suzuki H, Matsuoka M (2012). TDP-43 toxicity is mediated by the unfolded protein response-unrelated induction of C/EBP homologous protein expression. J. Neurosci. Res..

[27] Suzuki H, Matsuoka M (2013). The JNK/c-Jun signaling axis contributes to the TDP-43-induced cell death. Mol. Cell. Biochem..

[28] Thomson E, Ferreira-Cerca S, Hurt E (2013). Eukaryotic ribosome biogenesis at a glance. J. Cell Sci..

[29] Henras AK, Plisson-Chastang C, O’Donohue MF, Chakraborty A, Gleizes PE (2015). An overview of pre-ribosomal RNA processing in eukaryotes. Wiley Interdiscip. Rev. RNA.

[30] Drygin D (2011). Targeting RNA polymerase I with an oral small molecule CX-5461 inhibits ribosomal RNA synthesis and solid tumor growth. Cancer Res..

[31] Arabi A (2005). c-Myc associates with ribosomal DNA and activates RNA polymerase I transcription. Nat. Cell Biol..

[32] Grandori C (2005). c-Myc binds to human ribosomal DNA and stimulates transcription of rRNA genes by RNA polymerase I. Nat. Cell Biol..

[33] Grewal SS, Li L, Orian A, Eisenman RN, Edgar BA (2005). Myc-dependent regulation of ribosomal RNA synthesis during Drosophila development. Nat. Cell Biol..

[34] van Riggelen J, Yetil A, Felsher DW (2010). MYC as a regulator of ribosome biogenesis and protein synthesis. Nat. Rev. Cancer.

[35] Jalal C, Uhlmann-Schiffler H, Stahl H (2007). Redundant role of DEAD box proteins p68 (Ddx5) and p72/p82 (Ddx17) in ribosome biogenesis and cell proliferation. Nucleic Acids Res..

[36] Geissler V, Altmeyer S, Stein B, Uhlmann-Schiffler H, Stahl H (2013). The RNA helicase Ddx5/p68 binds to hUpf3 and enhances NMD of Ddx17/p72 and Smg5 mRNA. Nucleic Acids Res..

[37] Mizielinska S (2014). C9orf72 repeat expansions cause neurodegeneration in Drosophila through arginine-rich proteins. Science.

[38] Lopez-Gonzalez R (2016). Poly(GR) in C9ORF72-related ALS/FTD compromises mitochondrial function and increases oxidative stress and DNA damage in iPSC-derived motor neurons. Neuron.

[39] Freibaum BD (2015). GGGGCC repeat expansion in C9orf72 compromises nucleocytoplasmic transport. Nature.

[40] Haeusler AR (2014). C9orf72 nucleotide repeat structures initiate molecular cascades of disease. Nature.

[41] Hartmann H (2018). Proteomics and C9orf72 neuropathology identify ribosomes as poly-GR/PR interactors driving toxicity. Life Sci. Alliance.

[42] Coller HA (2000). Expression analysis with oligonucleotide microarrays reveals that MYC regulates genes involved in growth, cell cycle, signaling, and adhesion. Proc. Natl Acad. Sci. USA.

[43] Kim S, Li Q, Dang CV, Lee LA (2000). Induction of ribosomal genes and hepatocyte hypertrophy by adenovirus-mediated expression of c-Myc in vivo. Proc. Natl Acad. Sci. USA.

[44] Boon K (2001). N-myc enhances the expression of a large set of genes functioning in ribosome biogenesis and protein synthesis. EMBO J..

[45] Gomez-Roman N, Grandori C, Eisenman RN, White RJ (2003). Direct activation of RNA polymerase III transcription by c-Myc. Nature.

[46] Schlosser I (2003). A role for c-Myc in the regulation of ribosomal RNA processing. Nucleic Acids Res..

[47] Henning D, So RB, Jin R, Lau LF, Valdez BC (2003). Silencing of RNA helicase II/Gualpha inhibits mammalian ribosomal RNA production. J. Biol. Chem..

[48] Yang H (2003). Down-regulation of RNA helicase II/Gu results in the depletion of 18 and 28 S rRNAs in Xenopus oocyte. J. Biol. Chem..

[49] Calo E (2015). RNA helicase DDX21 coordinates transcription and ribosomal RNA processing. Nature.

[50] Zhang Y, Forys JT, Miceli AP, Gwinn AS, Weber JD (2011). Identification of DHX33 as a mediator of rRNA synthesis and cell growth. Mol. Cell Biol..

[51] Zonta E (2013). The RNA helicase DDX5/p68 is a key factor promoting c-fos expression at different levels from transcription to mRNA export. Nucleic Acids Res..

