# Supplementary material for: The proline–arginine repeat protein linked to C9-ALS/FTD causes neuronal toxicity by inhibiting the DEAD-box RNA helicase-mediated ribosome biogenesis
Source: Cell Death Dis. 2018 Sep 24;9(10):975. doi: 10.1038/s41419-018-1028-5 (PMC6155127; doi:10.1038/s41419-018-1028-5)
Supplement: Supplementary file 1 — Fig. S1 and Table S1 [file 41419_2018_1028_MOESM1_ESM.pdf]

Supplementary information

Fig. S1 Poly-PR binds to mature rRNA species.

a

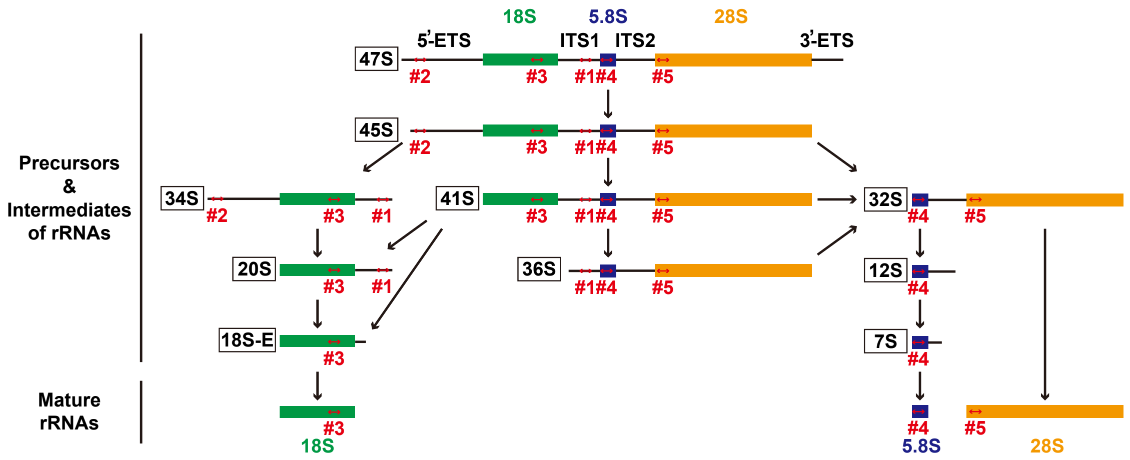

a Overview of pre-rRNA processing and priming sites used in RNA immunoprecipitation (RIP) assays. Major precursors and intermediates of mouse rRNA that are transcribed by RNA polymerase I are depicted. 5S rRNA is generated by RNA polymerase III (not shown here). Mature 18S, 5.8S, and 28S rRNA are separated by the internal transcribed spacer 1 (ITS1) and ITS2 and are flanked by the 5'-external transcribed spacer (5'-ETS) and 3'-ETS. Ribosomal RNA is initially transcribed as a full-length precursor (47S) that is rapidly processed to generate 45S pre-rRNA. Multiple precursors and intermediates are then endonucleolytically and exonucleolytically processed in a stepwise fashion. Priming sites used in the RIP assay are shown in red-letter. The annealing positions of primer sets-#1 and -#2 are located within ITS1 and 5'-ETS, respectively. The annealing positions of primer sets-#3, -#4, and -#5 are located within mature 18S, 5.8S, and 28S rRNA, respectively.

b

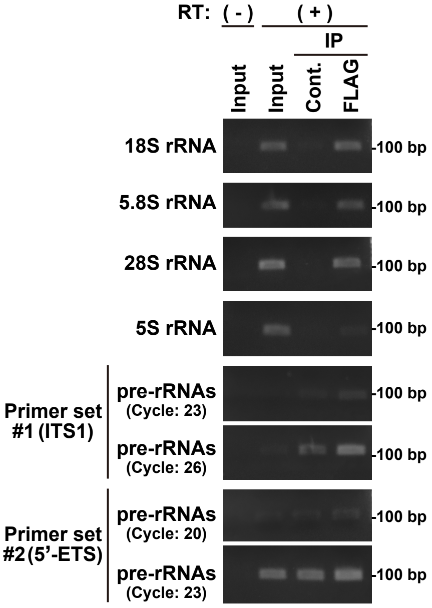

b FLAG-PR100 binds to 18S, 5.8S, 28S, and 5S rRNA, but not pre-rRNAs. NSC-34 cells were transfected with the FLAG-PR100-encoding vector. At 48 h after the transfection, the cell lysates were immunoprecipitated (IP) with normal mouse IgG (Cont.) or the FLAG antibody. Precipitates were then used for RIP assays. RT (-) was used as negative control to monitor the PCR amplification from genomic DNA. Mature 18S, 5.8S, and 28S rRNA were amplified with primer sets-#3, -#4, and -#5, respectively. Pre-rRNAs are amplified with the primer sets-#1 and -#2 (Fig. S1a). Mature 18S, 5.8S, 28S, and 5S rRNA, co-precipitated with FLAG-PR100, were amplified in the FLAG antibody immunoprecipitates, whereas the background binding of pre-rRNAs to FLAG-PR100 and control IgG gave rise to the nearly similar background amplification of pre-rRNAs in advanced PCR cycles using both primer sets-#1 and -#2.

Table S1

A summary of candidates of poly-PR-binding protein that may localize in the nucleolus

Band No.	Protein name	Accession	Total score	Sequence coverage (%)	No. of peptides matched
2	ATP-dependent RNA helicase A (Dhx9)	O70133	46.7	22.1	27
	Myb-binding protein 1A	Q7TPV4	35.8	17.8	19
5	Poly [ADP-ribose] polymerase 1	P11103	71.6	34.3	49
6	Nucleolar protein 14	Q8R3N1	4.2	5.0	3
7	DNA topoisomerase 1	Q04750	10.0	8.6	5
	**Nucleolar RNA helicase 2 (Ddx21)**	Q9JIK5	8.4	5.3	4
	Nucleolin	P09405	29.5	18.5	16
9	**ATP-dependent RNA helicase DDX18**	Q8K363	14.0	13.0	7
	Fragile X mental retardation protein 1 homolog	P35922	12.6	16.0	8
10	Nucleolar GTP-binding protein 1	Q99ME9	6.1	4.6	3
	**Probable ATP-dependent RNA helicase DDX17**	Q501J6	3.5	4.9	3
	**Probable ATP-dependent RNA helicase DDX5**	Q61656	5.3	6.5	4
	RNA-binding protein 14	Q8C2Q3	12.3	11.8	7
	Stress-70 protein, mitochondrial	P38647	28.7	28.9	17
11	H/ACA ribonucleoprotein complex subunit 4	Q9ESX5	3.1	6.9	2
	Non-POU domain-containing octamer-binding protein	Q99K48	14.4	22.0	8
12	Cell growth-regulating nucleolar protein	Q08288	4.0	9.3	2
13	Elongation factor 1-alpha 1	P10126	44.0	43.5	34
	KRR1 small subunit processome component homolog	Q8BGA5	7.3	11.8	4
14	Complement component 1 Q subcomponent-binding protein, mitochondrial	O35658	24.5	54.0	31
	mRNA turnover protein 4 homolog	Q9D0I8	10.6	25.9	5

Proteins in bold type belong to DEAD-box RNA helicases. Band numbers correspond to those in Figure 6a. A “total score” is a value for the level of confidence in the protein identification, calculated by ProteinPilot. It can be converted to a percentage confidence score using the following formula: ProtScore = -log(1-percentage confidence/100). As an approximate guide, ProteinPilot total scores give the following percentage levels of confidence; score >2 (>99% confidence), score >3 (>99.9% confidence). A “sequence coverage (%)” is a percentage of an identified protein that is covered by all matched peptides.
